# Silver Tip (*Camellia sinensis*) Extract Promotes Supersulfide Biosynthesis in Keratinocytes

**DOI:** 10.3390/ijms27104214

**Published:** 2026-05-09

**Authors:** Kento Kunihiro, Katsura Sano, Shogo Suzuki

**Affiliations:** ALBION Co., Ltd., 1-7-10 Ginza, Chuo-ku, Tokyo 104-0061, Japan; ka_maeda@albion.co.jp (K.S.); sh_suzuki@albion.co.jp (S.S.)

**Keywords:** *Camellia sinensis*, silver tip, keratinocytes, supersulfides, polyphenols

## Abstract

“Supersulfides” is a general term for compounds containing multiple sulfur atoms within their molecules. Owing to their potent anti-oxidant and anti-inflammatory activities, these species are promising ingredients in the field of cosmetics. In this study, we elucidated the effect of silver tip (*Camellia sinensis*) tea on supersulfide production in epidermal keratinocytes. Silver tip extract increased the fluorescence intensity of sulfane sulfur probe 4 (SSP4) in a concentration-dependent manner and promoted supersulfide production in keratinocytes. In particular, (−)-epicatechin gallate and (−)-epigallocatechin gallate exhibited high SSP4 fluorescence intensity, indicating that these are the active components. Mechanistic analysis using quantitative polymerase chain reaction revealed that silver tip extract promotes intracellular supersulfide production by regulating supersulfide-related metabolic factors (cysteinyl-tRNA synthetase 2, cystathionine β-synthase, cystathionine γ-lyase, solute carrier family 7 member 11, and nuclear factor E2-related factor 2). Furthermore, these compounds significantly increased the supersulfide levels by reacting with sodium sulfide, a hydrogen sulfide donor, in buffer solution, thereby catalytically enhancing supersulfide production. Overall, the results of this study indicate that silver tip extract, rich in polyphenols, regulates supersulfide metabolism in keratinocytes, suggesting its potential as an anti-aging ingredient for the skin.

## 1. Introduction

Supersulfides are molecular species possessing a structure in which sulfur atoms are linked, including hydropersulfides (RSSHs) and polysulfide species (RSS*n*R; *n* > 1, R = hydrogen, and alkyl or cyclic sulfurs) [1,2]. In living organisms, supersulfides exist as low-molecular-weight supersulfides and protein supersulfides [3]. Several enzymes, including cysteinyl-tRNA synthetase 2 (CARS2), cystathionine β-synthase (CBS), cystathionine γ-lyase (CSE), and 3-mercaptopyruvate sulfurtransferase (MPST), are reported to catalyze supersulfide biosynthesis [4,5,6]. Furthermore, intracellular supersulfides are considered to be regulated by the upregulation of solute carrier family 7 member 11 (SLC7A11; xCT) induced by nuclear factor E2-related factor 2 (NRF2) activation [7]. Supersulfide species such as cysteine persulfide (CysSSH), cysteine trisulfide (CysSSSCys), glutathione persulfide (GSSH), and glutathione trisulfide (GSSSG) have been previously reported [4,8,9,10]. Supersulfides are currently attracting widespread attention as a physiologically active metabolite synthesized by all living organisms. GSSSG increases mitochondrial membrane potential and promotes ATP production [7]. GSSSG also inhibits lipopolysaccharide-induced inflammation in retinal pigment epithelial and glial cells, prevents neurodegeneration in the spinal cord, protects the airways in viral and chronic lung diseases, and suppresses lipid peroxidation [2,11,12,13]. CysSSSCys is known to reduce lipid peroxidation and protect the heart from myocardial ischemia-reperfusion injury [14]. N-Acetylcysteine tetrasulfide inhibits inflammation-inducing signals via toll-like receptor signaling and exerts protective effects against endotoxin shock [15]. Therefore, considering their influence on various signal transduction processes, supersulfides are attracting attention in diverse research fields, including medicine, pharmacology, and biology. Importantly, supersulfide is an anti-oxidant with radical scavenging activity more potent than that of trolox or ascorbic acid, and participates in lifespan regulation by reducing endoplasmic reticulum stress and enhancing energy metabolism [16,17]. Hence, supersulfide may offer protective and anti-aging effects in the skin, the outermost layer constantly exposed to external stress. Recent research has revealed that supersulfides exist at high concentrations in the serum and are widely distributed throughout diverse tissues and bodily fluids, including the heart, hair, and semen [18,19]. However, very few studies have reported supersulfide detection within skin tissue. We recently reported the presence of supersulfide in both the epidermal and dermal layers of the skin. Functional analysis in epidermal keratinocytes revealed that supersulfides regulate keratinocyte cell migration, indicating their potential for inducing skin regeneration [20]. We also screened cosmetic ingredients that promote the biosynthesis of intracellular supersulfides in keratinocytes and observed that extracts from green tea and black tea may promote intracellular supersulfide biosynthesis in keratinocytes; however, the active components and underlying mechanisms remain unknown.

Tea (*Camellia sinensis*) is one of the most popular beverages globally and is widely used in food, cosmetics, and pharmaceuticals. Depending on the processing techniques used, tea is classified as green tea, white tea, yellow tea, oolong tea, black tea, and dark tea [21]. Tea grades are determined by the part of the plant used, with higher grades containing more buds [22]. Silver tip tea only uses buds without leaves, making it highly prized by connoisseurs and extremely rare compared to green tea [23]. Silver tip tea contains components similar to those of green tea, including catechins, alkaloids, and horbalones, and is particularly rich in (−)-epigallocatechin gallate (EGCG) and (−)-epicatechin gallate (ECG). Despite minimal differences in the chemical composition between silver tip tea and green tea, silver tip tea is reported to contain higher levels of theanine, amino acids, and flavonols [24]. Green tea undergoes a process called fixing, which destroys the tea leaf tissue and stops the activity of polyphenol oxidase, whereas silver tip tea undergoes the least processing compared with that of other teas, resulting in minimal changes to the plant components [24,25]. Furthermore, previous studies have reported various functional properties of green tea, including protection against photoaging of the skin, anti-aging effects, stress resistance, autophagy activation, and anti-oxidant activity [26,27,28,29]. However, the effects of silver tip tea extract on the skin remain unexplored. Therefore, in this study, we aimed to investigate the effects of silver tip tea extract on supersulfide production in keratinocytes and its potential for developing new cosmetic ingredients.

## 2. Results

### 2.1. Silver Tip Extract Produces Supersulfides in Keratinocytes

**Figure 1:**
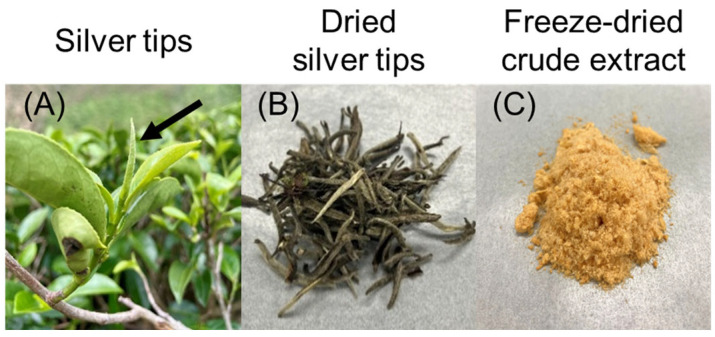
Silver tips and freeze-dried extract of *Camellia sinensis* (*C. sinensis*). (**A**) Silver tips are young leaves (leaf buds) in the process of unfolding. (**B**) Dried silver tips. (**C**) Freeze-dried material from the crude extract obtained from silver tips.

**Figure 2:**
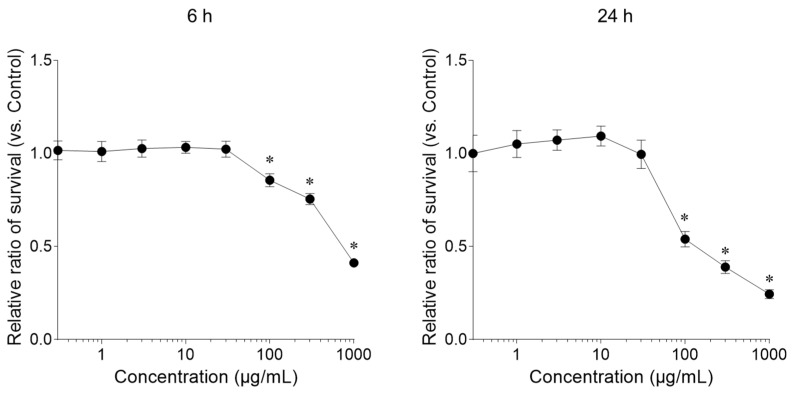
Effect of the silver tip extract on keratinocyte viability. Data are presented as mean ± standard deviation (SD) from three independent biological replicates (*n* = 3), each experiment consisting of 10 technical replicates per condition. * *p* < 0.05 vs. control using one-way ANOVA followed by Dunnett’s multiple comparison test.

In this study, a freeze-dried aqueous extract prepared from dried silver tips, consisting of unopened buds of *Camellia sinensis*, was used (Figure 1). First, we measured the effect of the silver tip extract on keratinocyte viability using the Cell Counting Kit-8. Cells treated with the silver tip extract for 24 h showed a significant decrease in viability at concentrations higher than 100 μg/mL compared with that in the control. However, cells treated with the silver tips extract for 6 h exhibited low cytotoxicity at concentrations below 100 μg/mL (Figure 2). Based on these results, subsequent experiments were conducted within a concentration range of 3–100 μg/mL.

**Figure 3:**
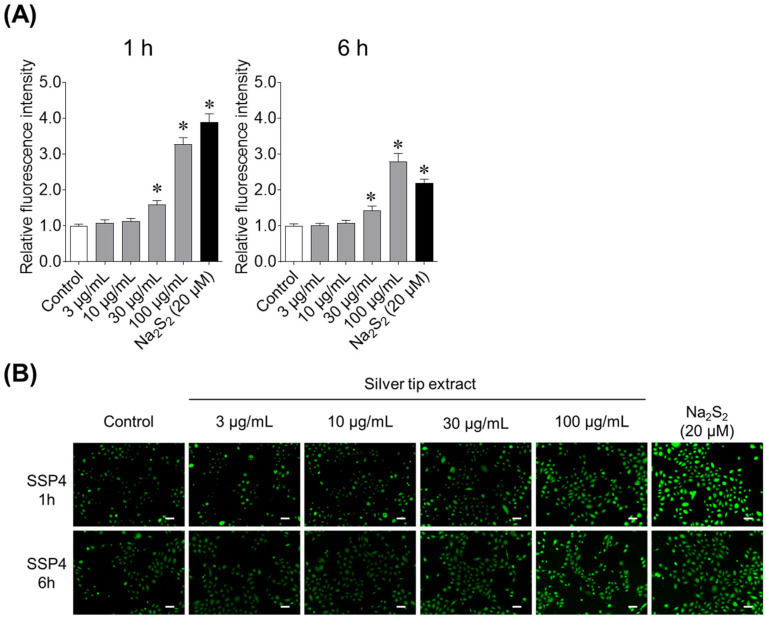
Effect of the silver tip extract on supersulfide production in keratinocytes in short-term (1 h) and long-term (6 h) studies. (**A**) The fluorescence intensity of the sulfane sulfur probe 4 (SSP4) was measured using a fluorescence plate reader. Data are presented as mean ± SD from three independent biological replicates (*n* = 3), each experiment consisting of 10 technical replicates per condition. * *p* < 0.05 vs. the control using one-way ANOVA followed by Dunnett’s multiple comparison test. (**B**) Fluorescence images (green) of SSP4 were captured using a fluorescence microscope. The scale bar represents 50 μm.

We then investigated the effect of the silver tip extract on supersulfide production in keratinocytes using the SSP4. Adding sodium disulfide (Na_2_S_2_), a supersulfide donor, to keratinocytes increased the fluorescence intensity of SSP4. Furthermore, the silver tip extract increased supersulfide production in keratinocytes in a concentration-dependent manner, with this effect observed at concentrations of 30 μg/mL or higher (Figure 3). The effect was comparable to that of Na_2_S_2_, the positive control. Furthermore, the supersulfide production-inducing effect of the silver tip extract was confirmed in both short-term (1 h) and long-term (6 h) tests.

### 2.2. Identification of the Active Substance in Silver Tip Extract Responsible for Supersulfide Synthesis in Keratinocytes

**Figure 4:**
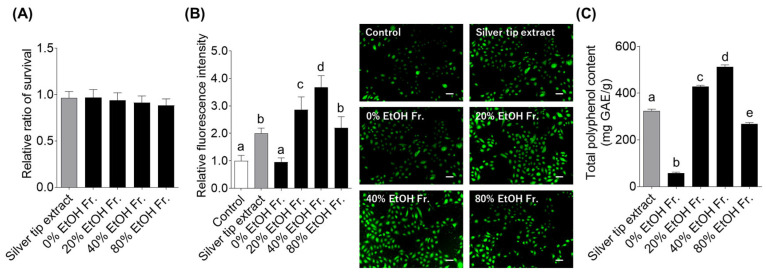
Cell viability effects, supersulfide production-inducing effects, and total polyphenol content of silver tip extract and its fractions obtained using DIAION HP-20 column chromatography. Cells were treated with 30 μg/mL silver tip extract or each fraction for 6 h. (**A**) Cell viability was measured using Cell Counting Kit-8. Data are presented as mean ± SD from three independent biological replicates (*n* = 3), each experiment consisting of 10 technical replicates per condition. (**B**) Supersulfide production was measured based on fluorescence intensity and fluorescence imaging using SSP4. Data are presented as mean ± SD from three independent biological replicates (*n* = 3), each experiment consisting of 10 technical replicates per condition. The means within columns with the same superscript letters are not significantly different (*p* < 0.05). The scale bar represents 50 μm. (**C**) The total polyphenol content was determined using the Folin–Ciocalteu assay. Data are presented as mean ± SD from three independent measurements. The means within columns with the same superscript letters are not significantly different (*p* < 0.05).

To identify compounds that activate supersulfide production in keratinocytes, the silver tip extract was subjected to column chromatography for obtaining 0%, 20%, 40%, and 80% EtOH fractions. The yields for each fraction were 44.3%, 19.0%, 27.7%, and 1.3%, respectively. We investigated the effects of these fractions on cell viability and SSP4 fluorescence in keratinocytes. First, we confirmed that none of the fractions induced cytotoxicity in keratinocytes (Figure 4A). Next, when the supersulfide production effect of the fractions was measured using SSP4, the 20%, 40%, and 80% EtOH fractions showed strong SSP4 fluorescence intensity, whereas the 0% EtOH fraction showed no difference when compared with the control (Figure 4B). In particular, the fluorescence intensity of SSP4 was significantly higher in the 20% and 40% EtOH fractions. As polyphenols are the major components of *C. sinensis* [30], the total polyphenol content was quantified. The result showed that 20% and 40% EtOH fractions contained abundant polyphenols (Figure 4C).

**Figure 5:**
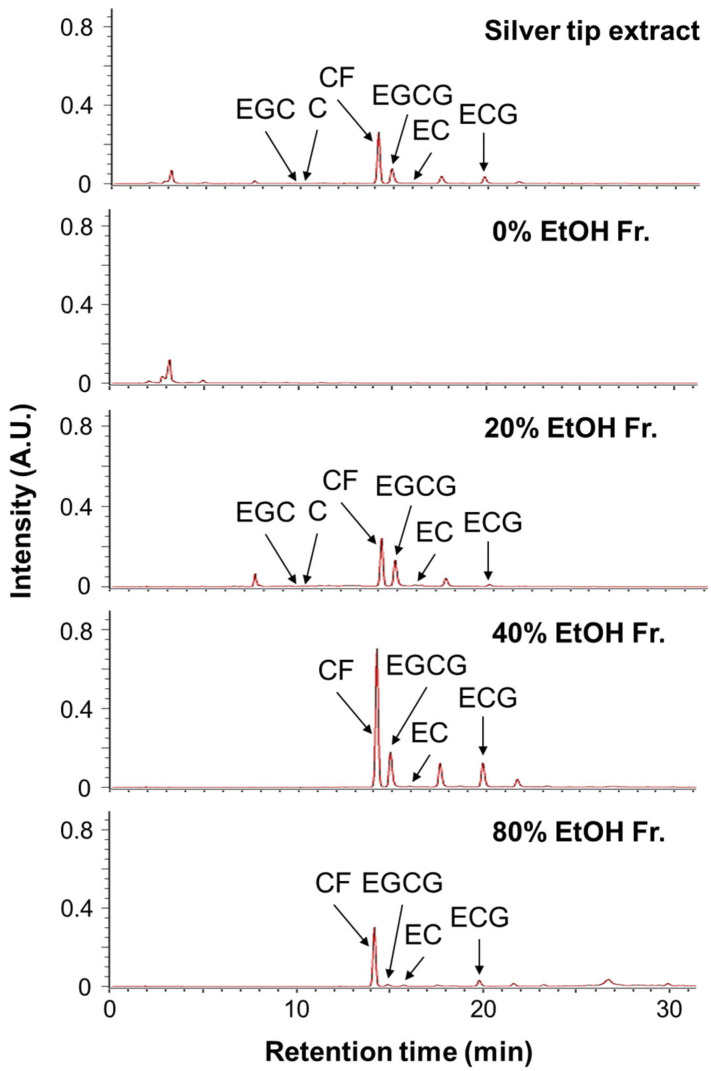
High-performance liquid chromatography (HPLC) chromatogram of the silver tips extract and its fractions. Detection, PDA detector (280 nm); EGC, (−)-epigallocatechin; C, (+)-catechin; CF, caffeine; EGCG, (−)-epigallocatechin gallate; EC, (−)-epicatechin; ECG, (−)-epicatechin gallate.

**Figure 6:**
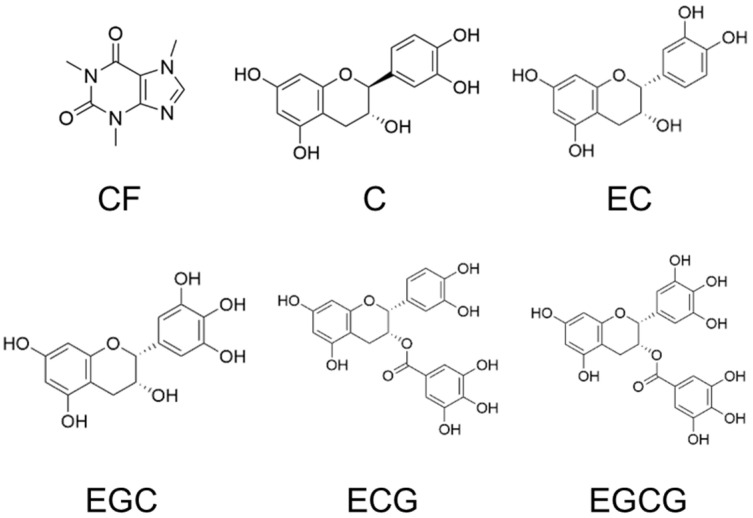
Structures of compounds in the silver tip extract. CF, caffeine; C, (+)-catechin; EC, (−)-epicatechin; EGC, (−)-epigallocatechin; ECG, (−)-epicatechin gallate; EGCG, (−)-epigallocatechin gallate.

High-performance liquid chromatography (HPLC) analysis was performed to identify the polyphenols in the silver tip extract. The HPLC chromatograms of the silver tip extract and its fractions at 280 nm are shown in Figure 5. The detected peaks were identified as the non-polyphenolic compound caffeine (CF) and the polyphenols (+)-catechin (C), (−)-epicatechin (EC), (−)-epigallocatechin (EGC), (−)-epicatechin gallate (ECG), and (−)-epigallocatechin gallate (EGCG) (Figure 6). Quantitative analysis using HPLC revealed that the amounts of CF, C, EC, EGC, ECG, and EGCG in the silver tip extract were 224.3, 11.0, 9.5, 20.2, 56.6, and 110.7 mg/g, respectively (Table 1). Furthermore, based on the yield of the silver tip extract, the contents of CF, C, EC, EGC, ECG, and EGCG in dried silver tips were calculated as 88.0, 4.3, 3.7, 7.9, 22.2, and 43.4 mg/g, respectively (Table 1). These results indicated that CF is the major component of the silver tip extract and that gallate-type catechins such as ECG and EGCG are the major polyphenols.

**Figure 7:**
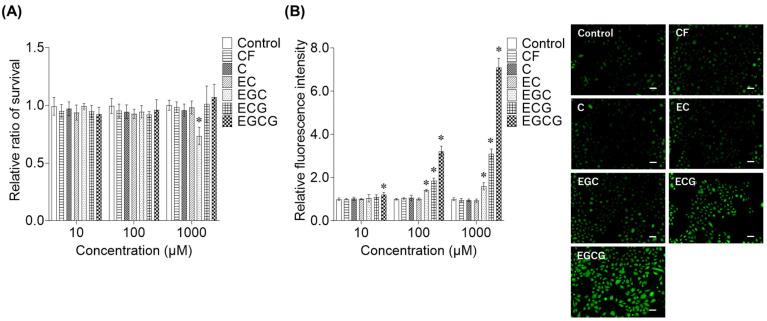
Cell viability and supersulfide production effects of compounds in the silver tip extract. Cells were treated with each compound at concentrations ranging from 10 to 1000 μM for 6 h. (**A**) Cell viability was measured using the Cell Counting Kit-8. Data are presented as mean ± SD from three independent biological replicates (*n* = 3), each experiment consisting of 10 technical replicates per condition. (**B**) Supersulfide production was measured based on fluorescence intensity and fluorescence imaging using SSP4. Data are presented as mean ± SD from three independent biological replicates (*n* = 3), each experiment consisting of 10 technical replicates per condition. * *p* < 0.05 vs. control using one-way ANOVA followed by Dunnett’s multiple comparison test. The fluorescence images of each compound using SSP4 are representative images obtained at a concentration of 100 μM. Scale bar represents 50 μm. CF: caffeine; C: (+)-catechin; EC: (−)-epicatechin; EGC: (−)-epigallocatechin; ECG: (−)-epicatechin gallate; EGCG: (−)-epigallocatechin gallate.

To identify the active components responsible for supersulfide production in the silver tip extract, cell viability and SSP4 assays were conducted with each compound. The cell survival rates in keratinocytes treated with CF, C, EC, ECG, and EGCG revealed no toxicity within the tested concentration range of 10–1000 μM; however, EGC caused an approximately 30% reduction in cell numbers at 1000 μM (Figure 7A). The supersulfide production effects of each compound were examined using SSP4, which revealed high activity in the order of EGC, ECG, and EGCG (Figure 7B). In particular, gallate-type catechins such as ECG and EGCG showed potent supersulfide-generating activity compared with that of other catechins.

### 2.3. Analysis of the Supersulfide Synthesis Mechanism Activated by the Silver Tip Extract in Keratinocytes

**Figure 8:**
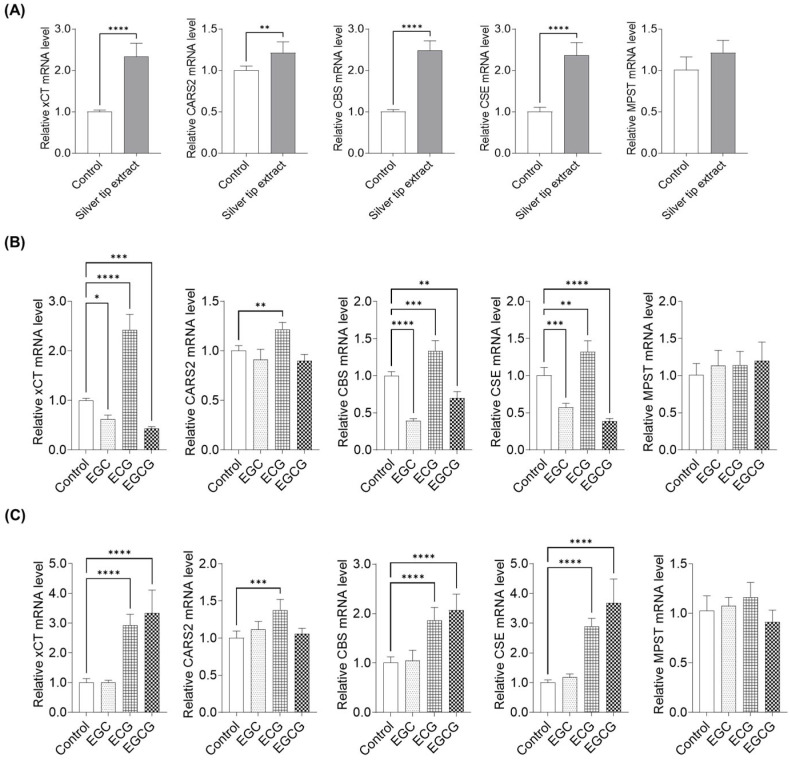
Effects of the silver tip extract and compounds on gene expression related to supersulfide metabolism in keratinocytes. Cells were treated for 6 h with silver tip extract, (−)-epigallocatechin (EGC), (−)-epicatechin gallate (ECG), or (−)-epigallocatechin gallate (EGCG), followed by qPCR analysis of solute carrier family 7 member 11 (xCT), which mediates the uptake of supersulfide substrates, and the supersulfide-synthetic enzymes cysteinyl-tRNA synthetase 2 (CARS2), cystathionine β-synthase (CBS), cystathionine γ-lyase (CSE), and 3-mercaptopyruvate sulfurtransferase (MPST). mRNA expression levels were normalized to GAPDH expression and calculated relative to those in control cells. (**A**) Gene expression after treatment with 30 µg/mL silver tip extract. Data are presented as mean ± SD from three independent biological replicates (*n* = 3), each experiment consisting of 6 technical replicates per condition. * *p* < 0.05 vs. control using unpaired two-tailed Student’s *t*-test. (**B**) Gene expression after treatment with EGC, ECG, or EGCG at 100 µM. (**C**) Gene expression after treatment with EGC, ECG, or EGCG at concentrations adjusted to match their levels in 30 µg/mL silver tip extract. Data are presented as mean ± SD from three independent biological replicates (*n* = 3), each experiment consisting of 6 technical replicates per condition. **** *p* < 0.0001, *** *p* < 0.001, ** *p* < 0.01, * *p* < 0.05 vs. control using one-way ANOVA followed by Dunnett’s multiple comparison test.

To investigate the mechanism underlying silver tip extract-induced supersulfide production in keratinocytes, we examined the expression of genes related to supersulfide biosynthesis using qPCR after treatment with the extract, EGC, ECG, and EGCG. The target genes included solute carrier family 7 member 11 (xCT), which mediates the uptake of supersulfide substrates, as well as the supersulfide-synthetic enzymes cysteinyl-tRNA synthetase 2 (CARS2), cystathionine β-synthase (CBS), cystathionine γ-lyase (CSE), and 3-mercaptopyruvate sulfurtransferase (MPST). Treatment with silver tip extract increased the expression of xCT, CARS2, CBS, and CSE. In particular, the expression levels of xCT, CBS, and CSE increased by 2.3-fold, 2.5-fold, and 2.4-fold, respectively, compared with those in the control. In contrast, silver tip extract did not affect MPST expression (Figure 8A). Next, EGC, ECG, and EGCG, which showed increased SSP4 fluorescence intensity, were treated at a concentration of 100 µM, and gene expression was analyzed using qPCR. ECG induced upregulation of xCT, CARS2, CBS, and CSE, whereas EGC and EGCG decreased the expression of these genes (Figure 8B). As shown in Figure 3, silver tip extract promoted supersulfide synthesis at concentrations greater than 30 µg/mL. Therefore, we adjusted the concentrations of EGC, ECG, and EGCG to match their levels in 30 µg/mL of silver tip extract (2 µM EGC, 3.8 µM ECG, and 7.3 µM EGCG) and confirmed the gene expression effects. The results showed that ECG and EGCG, which markedly enhanced SSP4 fluorescence intensity, significantly increased the expression of xCT, CBS, and CSE (Figure 8C).

**Figure 9:**
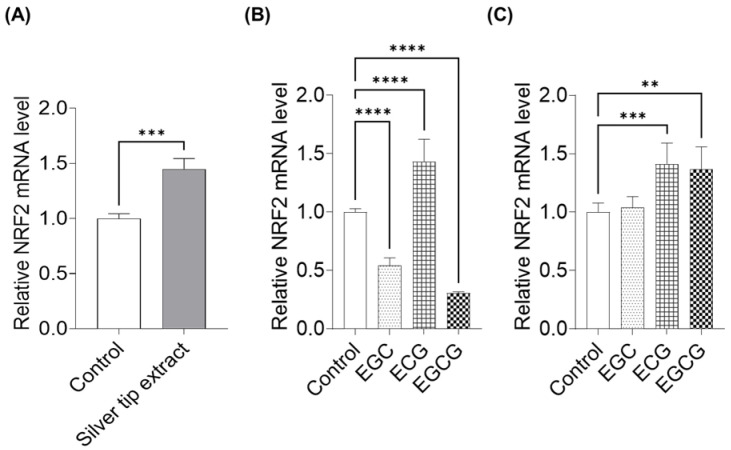
Effects of the silver tip extract and compounds on nuclear factor erythroid 2-related factor 2 (NRF2) expression in keratinocytes. Cells were treated for 6 h with silver tip extract, EGC, ECG, or EGCG, followed by qPCR analysis of NRF2. mRNA expression levels were normalized to GAPDH expression and calculated relative to those in control cells. (**A**) Gene expression after treatment with 30 µg/mL silver tip extract. Data are presented as mean ± SD from three independent biological replicates (*n* = 3), each experiment consisting of 6 technical replicates per condition. *** *p* < 0.001 vs. control using unpaired two-tailed Student’s *t*-test. (**B**) Gene expression after treatment with EGC, ECG, or EGCG at 100 µM. (**C**) Gene expression after treatment with EGC, ECG, or EGCG at concentrations adjusted to match their levels in 30 µg/mL silver tip extract. Data are presented as mean ± SD from three independent biological replicates (*n* = 3), each experiment consisting of 6 technical replicates per condition. **** *p* < 0.0001, *** *p* < 0.001, ** *p* < 0.01 vs. control using one-way ANOVA followed by Dunnett’s multiple comparison test.

We also examined the expression of NRF2, a transcriptional regulator involved in redox and sulfur metabolism. Treatment with the silver tip extract increased NRF2 mRNA expression in keratinocytes (Figure 9A). When individual catechins were applied at a concentration of 100 µM, ECG increased NRF2 expression, whereas EGC and EGCG reduced NRF2 mRNA levels (Figure 9B). In contrast, when catechins were adjusted to concentrations equivalent to those present in 30 µg/mL of the silver tip extract, both ECG and EGCG significantly increased NRF2 expression, whereas EGC showed no effect (Figure 9C). These results exhibited a trend similar to that observed for the expression of sulfur metabolism-related genes.

**Figure 10:**
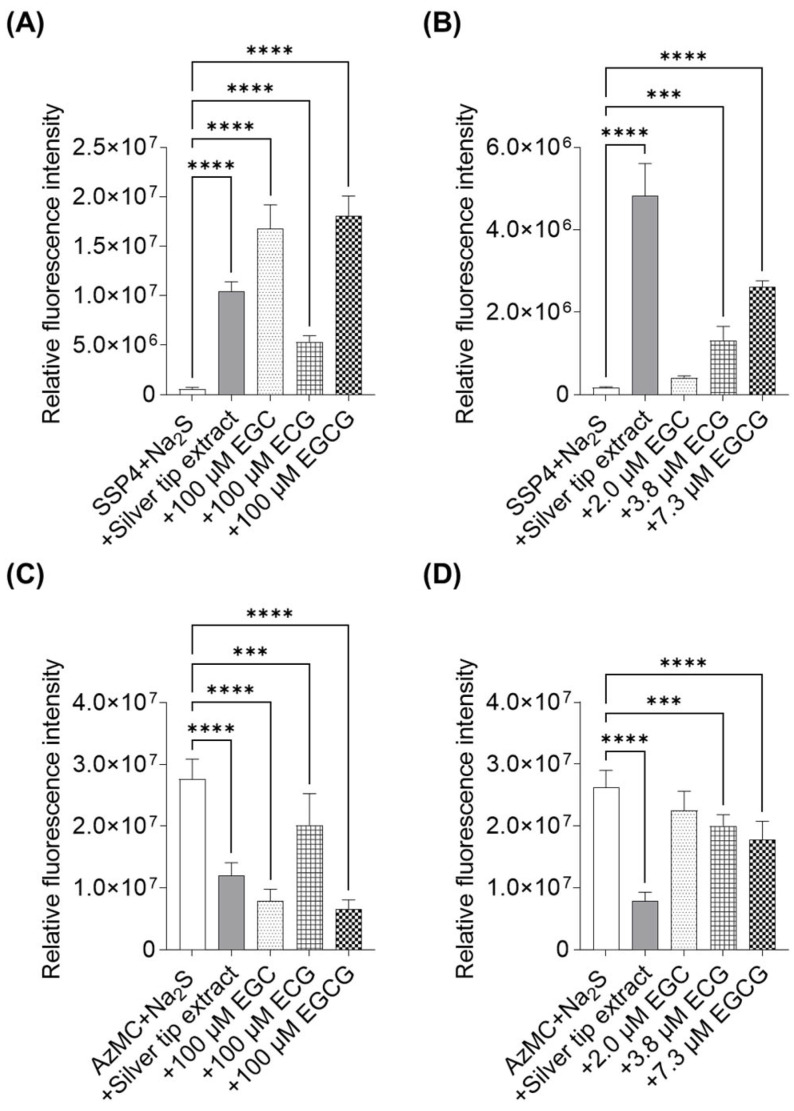
Effects of the silver tip extract and its compounds on sulfur metabolism. (**A**,**C**) Fluorescence intensity of the sulfane sulfur probe 4 (SSP4) and 7-azido-4-methylcoumarin (AzMC) after 6 h of incubation with H_2_S and 30 µg/mL silver extract, or 100 µM of EGC, ECG, or EGCG. (**B**,**D**) Fluorescence intensity of SSP4 and AzMC after 6 h incubation with H_2_S and 30 µg/mL silver extract, EGC, ECG, or EGCG at concentrations equivalent to those in the extract. Data are presented as mean ± SD from three independent measurements. **** *p* < 0.0001, *** *p* < 0.001 vs. sample-free condition using one-way ANOVA followed by Dunnett’s multiple comparison test. EGC; (−)-epigallocatechin, ECG; (−)-epicatechin gallate, EGCG; (−)-epigallocatechin gallate.

Furthermore, we verified the chemical generation of polysulfides by incubating silver tip extract and its compounds with H_2_S (prepared as Na_2_S), and assessed the effects on sulfur metabolism using SSP4 and AzMC. The silver tip extract increased SSP4 fluorescence intensity in the presence of H_2_S. EGC, ECG, and EGCG, prepared at 100 µM, also exhibited a marked increase in SSP4 fluorescence compared with that in the control, with EGC and EGCG showing the strongest effects (Figure 10A). In contrast, at concentrations equivalent to those in 30 µg/mL silver tip extract (2 µM EGC, 3.8 µM ECG, and 7.3 µM EGCG), only ECG and EGCG significantly enhanced SSP4 fluorescence (Figure 10B). Although EGC showed a tendency to increase SSP4 fluorescence, the difference was not statistically significant. Notably, all samples that affected SSP4 fluorescence also reduced AzMC fluorescence, indicating that their contribution to supersulfide formation was associated with H_2_S degradation (Figure 10C,D).

## 3. Discussion

Supersulfides are sulfur-linked molecular species composed of multiple concatenated sulfur atoms and exert a wide range of physiological functions. Previous studies have reported that supersulfides act as potent redox signaling molecules, converting chemical stimuli into biological responses and regulating key cellular processes such as energy metabolism, oxidative stress management, signal transduction, and cellular development [3]. Supersulfides are characterized by strong anti-oxidant activity, which exceeds that of trolox, a representative vitamin E derivative [16]. The skin, the largest organ of the human body and its outermost layer, is constantly exposed to external stressors [31]. Therefore, we hypothesized that supersulfides play an essential role in maintaining skin homeostasis. Our previous work demonstrated that supersulfides such as cysteine hydropersulfide (CysSSH) and glutathione hydropersulfide (GSSH) are present in the skin and regulate keratinocyte migration [20]. Our findings in this study indicate that silver tip tea extract may modulate supersulfide synthesis in keratinocytes. Our data demonstrate that the silver tip extract promotes supersulfide production by keratinocytes in a concentration-dependent manner. Furthermore, the fluorescence intensity of sulfane sulfur probe 4 (SSP4) in fractions obtained using column chromatography correlated with polyphenol content, suggesting that these components may contribute to supersulfide synthesis in keratinocytes. Interestingly, among catechins, (−)-epicatechin gallate (ECG) and (−)-epigallocatechin gallate (EGCG) markedly increased SSP4 fluorescence, indicating that gallate-type catechins are the likely active components responsible for inducing supersulfide production in keratinocytes. Previous studies have reported that natural extracts from blueberry, bilberry, cranberry, and matcha induce supersulfide formation in human embryonic kidney cells, with polyphenols including cyanidin, quercetin, rosmarinic acid, resveratrol, (−)-epigallocatechin (EGC), and EGCG contributing to these effects [32,33]. Our findings are consistent with these reports and further support the role of catechins as key active compounds. He et al. reported that among various catechins, EGCG demonstrates the highest anti-oxidant activity [34]. Fujimura et al. proposed that both the galloyl moiety and hydroxylation pattern of the B-ring determine the biological activity of catechins [35]. Our results also showed that the fluorescence intensity of SSP4 was correlated with the number of hydroxyl groups and the presence of galloyl groups. Notably, EGC increased SSP4 fluorescence intensity at concentrations above 100 μM; however, exposure to 1000 μM EGC also induced moderate cytotoxicity in keratinocytes. Given that EGC possesses multiple free phenolic hydroxyl groups but lacks a galloyl ester moiety, it is likely more susceptible to auto-oxidation under high-concentration conditions, which may result in enhanced generation of reactive oxygen species [36]. Therefore, the elevated SSP4 fluorescence observed at high EGC concentrations may, at least in part, reflect a cellular stress–associated response rather than selective supersulfide production. Caffeine, which lacks hydroxyl groups in its molecular structure, showed no variation in SSP4 fluorescence intensity. Among catechins, Catechin and (−)-epicatechin, which lack pyrogallol structures or galloyl groups, also showed no effect on supersulfide production. In contrast, EGCG, which contains both a galloyl group and a pyrogallol structure in its B-ring, exhibited a significantly stronger supersulfide production effect compared with that of EGC or ECG, each of which possesses either the pyrogallol structure or galloyl group. These results indicate that EGCG is the active component in silver tip extract responsible for promoting supersulfide generation.

Enzymes such as cysteinyl-tRNA synthetase 2 (CARS2), cystathionine β-synthase (CBS), cystathionine γ-lyase (CSE), and 3-mercaptopyruvate sulfurtransferase (MPST) are reported to be involved in supersulfide synthesis [37]. Furthermore, the cystine transporter xCT, encoded by solute carrier family 7 member 11 (SLC7A11), is known to increase intracellular persulfide levels [7,38]. The expression of xCT correlates with that of NRF2, a redox-sensitive transcription factor, and NRF2 has been reported to regulate xCT expression [38]. To explore the mechanism by which the silver tip extract promotes supersulfide synthesis in keratinocytes, we examined the expression of these genes. Silver tip extract increased the expression of NRF2, xCT, CARS2, CBS, and CSE. xCT produces supersulfide by relying on cystine (CysSSCys) uptake [39]. CARS2 catalyzes CysSSH formation from cysteine (CysSH) as the substrate, and converts it to other supersulfides such as GSSH and hydrogen disulfide (H_2_S_2_) [4,5]. CBS and CSE generate reactive sulfur species, such as CysSSH from CysSSCys, which then produce GSSH [40]. Previous studies indicate that activation of NRF2 increases xCT expression, which in turn is associated with enhanced expression of the supersulfide biosynthetic enzymes CBS and CSE [41]. Therefore, our data suggest that the silver tip extract promotes supersulfide synthesis in keratinocytes by upregulating synthetic enzymes such as CARS2, CBS, and CSE. Furthermore, the observed increases in NRF2 and xCT expressions suggest that activation of NRF2 influences CysSSCys uptake and subsequent CysSH levels, which may amplify supersulfide production via modulation of enzyme expression. In future studies, the effects of silver tip extract on CysSH and CysSSCys levels in keratinocytes need to be clarified, and its detailed impact on sulfur metabolism needs to be elucidated.

We also examined the effects of catechins involved in supersulfide synthesis within keratinocytes and analyzed their impact on sulfur metabolism-related gene expression to elucidate the mechanism at the compound level. In SSP4 assays, 100 µM of EGC, ECG, and EGCG significantly promoted intracellular supersulfide production. Accordingly, we analyzed supersulfide synthesis-related gene expression following treatment with EGC, ECG, and EGCG. Our results showed that ECG increased the expression of NRF2, xCT, CARS2, CBS, and CSE, whereas EGC and EGCG unexpectedly decreased the expression of these genes. Previous studies have also reported that EGCG catalytically oxidizes hydrogen sulfide (H_2_S) to generate polysulfides [33]. Therefore, we mixed sodium sulfide (Na_2_S), a hydrogen sulfide donor, with silver tip extract or individual catechins in buffer and measured the fluorescence from SSP4 and 7-azido-4-methylcoumarin (AzMC). Notably, EGC and EGCG markedly increased the SSP4 fluorescence and decreased AzMC fluorescence in buffer, suggesting catalytic conversion of H_2_S into supersulfides. Because these compounds synthesize supersulfides through non-enzymatic chemical reactions, negative feedback on NRF2, xCT, CBS, and CSE expression may occur to balance excessive supersulfide accumulation. In fact, the concentrations of catechins in the silver tip extract that enhanced SSP4 fluorescence were much lower than 100 µM. Therefore, we investigated gene expression and sulfur metabolism at concentrations equivalent to those in 30 µg/mL extract. Therefore, gallate-type catechins EGCG and ECG significantly increased the NRF2, xCT, CBS, and CSE expression, accompanied by increased SSP4 fluorescence and decreased AzMC fluorescence. Previous studies have also proposed that polyphenols undergo autoxidation to form semiquinone radicals, which act as oxidants for H_2_S. The semiquinone radical oxidizes hydrosulfide anions (HS^−^) to generate thiyl radicals (RS•). These RS• then combine to form persulfides or polysulfides. This reaction does not involve superoxide or hydrogen peroxide; instead, the semiquinone radical itself serves as the primary oxidizing agent. Polyphenols continuously cycle between hydroquinone, semiquinone, and quinone states, thus enabling sustained H_2_S oxidation and polysulfide formation [32]. Collectively, these findings suggest that the silver tip extract may promote supersulfide synthesis via at least two complementary pathways: (i) NRF2-dependent upregulation of xCT associated with enhanced substrate uptake, along with activation of enzymatic pathways involving increased expression of CBS and CSE; and (ii) putative chemical catalysis mediated by gallate-type catechins. Furthermore, ECG and EGCG were identified as the principal active components responsible for this effect. Supersulfides have been reported to exert potent anti-oxidant and cytoprotective effects [15,16], and therefore, the increased production of supersulfides in keratinocytes induced by silver tip extract suggests a potential contribution to anti-aging effects in the skin.

Extracts of *Camellia sinensis* have been widely used in anti-aging cosmetic products [42], direct experimental evidence demonstrating that the enhancement of supersulfide production in keratinocytes leads to improvement of skin aging remains limited. In addition, the major active components identified in this study, including ECG and EGCG, are generally known to exhibit relatively low skin permeability [43]. Therefore, optimization of cosmetic formulations—such as liposomal encapsulation, emulsions, or the use of penetration enhancers—will be essential to maximize their biological efficacy in the epidermis. Furthermore, this study did not evaluate the protein expression levels or enzymatic activities of supersulfide biosynthetic enzymes, nor did it fully recapitulate key physiological conditions of human skin, including pH gradients and the influence of cutaneous microbiota. Future studies should investigate how treatment with silver tip extract modulates both the expression and activity of supersulfide biosynthetic enzymes in keratinocytes and how these changes contribute to skin protection and potential anti-aging effects. In addition, ex vivo studies using excised human skin, as well as in vivo evaluations, will be required to elucidate the relationship between silver tip extract-induced changes in supersulfide levels, skin homeostasis, and overall skin health.

## 4. Materials and Methods

### 4.1. Plant Materials and Extraction

Dried silver tips (*Camellia sinensis*) were purchased from Macduff (C) Estate—Tea Factory (Lindula, Sri Lanka). Dried silver tips (70 g) were then extracted with distilled water (1400 mL) at 100 °C for 1 h. The resulting extract was filtered, evaporated, and lyophilized to obtain a crude extract (silver tip extract, 27.7 g).

### 4.2. Cell Culture

Normal human epidermal keratinocytes (NHEKs) were obtained from Thermo Fisher Scientific (Waltham, MA, USA) and cultured in EpiLife medium (Thermo Fisher Scientific) supplemented with 1% human keratinocyte growth supplement (HKGS). Cells were maintained at 37 °C in a humidified incubator with 5% CO_2_, according to the supplier’s protocol.

### 4.3. Cell Viability Assay

Cell viability was measured using the Cell Counting Kit-8 (Dojindo, Kumamoto, Japan). NHEK cells were seeded (1 × 10^4^ cells/well) in 96-well plates and cultured overnight. The cells were then treated with samples for 6 h or 24 h, and incubated with CCK-8 solution at 37 °C for 2 h. The absorbance at 450 nm was measured using a SpectraMax i3x instrument (Molecular Devices, San Jose, CA, USA). The data were calculated as absorbance values relative to those obtained in the absence of a sample.

For all cell-based experiments in this study, samples were dissolved in 20% (*v*/*v*) dimethyl sulfoxide (DMSO) and subsequently diluted with culture medium prior to treatment. The final concentration of DMSO was adjusted to be identical across all experimental groups and maintained at a non-cytotoxic level.

### 4.4. Sulfane Sulfur Probe 4 (SSP4)

NHEK cells were seeded (1 × 10^4^ cells/well) in 96-well black plates and cultured overnight. The cells were treated with samples for 1 h or 6 h, washed with Hanks’ Balanced Salt Solution (Thermo Fisher Scientific), and stained at 37 °C for 15 min with 20 μM SSP4 (Dojindo) and 0.5 mM cetyltrimethylammonium bromide (Fujifilm Wako Pure Chemicals, Osaka, Japan). Fluorescence images were captured using a BZ-X700 fluorescence microscope (Keyence, Osaka, Japan); the fluorescence intensity (em/ex, 482/515 nm) was determined using a SpectraMax i3x system.

### 4.5. Quantitative Polymerase Chain Reaction (qPCR) Assay

Total RNA was extracted using the TRI reagent (Molecular Research Center, Cincinnati, OH, USA) according to the manufacturer’s instructions. cDNA was synthesized through reverse transcription using the PrimeScript RT reagent kit (Takara Bio, Kusatsu, Japan). Total mRNA was quantified using a NanoDrop spectrophotometer (Thermo Fisher Scientific). qPCR was performed using a LightCycler 96 PCR system (Roche, Basel, Switzerland) and Luna Universal qPCR Master Mix (New England Biolabs, Ipswich, MA, USA). The target genes and corresponding primer sequences used were as follows: GAPDH, forward primer (F): 5′-GAGCCACATCGCTCAGACAC-3′ and reverse primer (R): 5′-TTGCCATGGGTGGAATCATA-3′; CARS2, F: 5′-GAAGCCGCCTCCTGGTATAG-3′ and R: 5′-TGCTGCATCCAAAAACCTTGG-3′; CBS, F: 5′-GTTAACACATGGCTTCCTAA-3′and R: 5′-CTCTCTTTTGCCTTTAATCC-3′; CSE, F: 5′-ATGGATGATGTGTATGGAGGTACAA-3′ and R: 5′-GTGCACAGCCTTCAATGTCAA-3′; MPST, F: 5′-CCAGGTACCGTGAACATCCC-3′ and R: 5′-ATGTACCACTCCACCCAGGA-3′; xCT, F: 5′-TGAAATCCCTGAACTTGCGAT-3′ and R: 5′- TCTGGATCCGGGCGCT-3′; and NRF2, F: 5′- TCAGCCAGCCCAGCACATCC-3′ and R: 5′-TCTGCGCCAAAAGCTGCATGC-3′. The measured data were analyzed using the delta threshold cycle method, and the expression of each gene was normalized to that of GAPDH, as described in a previous study [20].

### 4.6. Fractionation

The silver tip extract (1.0 g) was dissolved in distilled water and applied to a DIAION HP-20 column (Mitsubishi Chemical, Tokyo, Japan; 30 g; 150 mm × 40 mm i.d.) equilibrated with distilled water. The column was then eluted with an H_2_O-ethanol (EtOH) solvent system (100 mL) and four fractions, namely 0% EtOH Fr. (442.7 mg), 20% EtOH Fr. (189.6 g), 40% EtOH Fr. (277.4 mg), and 80% EtOH Fr. (13.3 mg), were obtained.

### 4.7. Determination of the Total Polyphenol Content

Total polyphenol levels were measured using the Folin–Ciocalteu assay [44]. Briefly, the silver tip extract and each fraction were diluted to 0.25 mg/mL in 50% methanol (MeOH) to prepare the sample solution. An aliquot of 80 µL was mixed with 160 µL of 1 N Folin–Ciocalteu reagent (Sigma Aldrich, St. Louis, MO, USA), followed by adding 400 µL of 1 N NaOH (Fujifilm Wako Pure Chemicals). After incubating the reaction mixture for 15 min at room temperature, the samples were centrifuged at 20,000× *g* for 5 min at 4 °C. The clear supernatant was transferred to a 96-well plate, and absorbance was measured at 734 nm using a SpectraMax i3x microplate reader. Total polyphenol content was expressed as mg gallic acid equivalents per gram of sample.

### 4.8. High-Performance Liquid Chromatography (HPLC) Analysis

The silver tip extract and each fraction were prepared in 50% MeOH at a concentration of 1 mg/mL and filtered using a 0.45 µm PTFE syringe filter (Cytiva, Tokyo, Japan). The standard compounds, (−)-epigallocatechin (EGC, Tokyo Chemical Industry, Tokyo, Japan), (+)-catechin (C, Fujifilm Wako Pure Chemicals), caffeine (CF, Fujifilm Wako Pure Chemicals), (−)-epigallocatechin gallate (EGCG, Adipogen Life Sciences, Liestal, Switzerland), (−)-epicatechin (EC, Fujifilm Wako Pure Chemicals), and (−)-epicatechin gallate (ECG, Fujifilm Wako Pure Chemicals), were prepared in 50% MeOH at concentrations ranging from 1 to 100 μg/mL.

Qualitative and quantitative analyses were performed on a Waters 2695 Alliance HPLC system (Waters, Milford, MA, USA) equipped with a Waters 2998 Photodiode Array (PDA) Detector (Waters) and an ODS-3 column (5 μm, 150 mm × 4.6 mm i.d., GL Sciences, Tokyo, Japan) using an MeOH-sodium dihydrogen phosphate (NaH_2_PO_4_, Fujifilm Wako Pure Chemicals) solvent system. Solution A was MeOH, solution B was 10 mM NaH_2_PO_4_, and the analysis was performed using a gradient of 10–50% solution A over 0–30 min. The flow rate was 1 mL/min, the column oven temperature was 40 °C, and the detection wavelength was 280 nm. Compounds were identified by directly comparing the HPLC chromatograms with those of the standards.

### 4.9. Measurement of Hydrogen Sulfide (H_2_S) and SSP4 Fluorescence Intensity in the Buffer

Effects on sample H_2_S and polysulfides were evaluated based on the fluorescence intensities of 7-azido-4-methylcoumarin (AzMC) and SSP4, respectively. AzMC is a fluorogenic probe for H_2_S in which the aromatic azide moiety is selectively reduced in the presence of H_2_S to generate the fluorescent compound 7-amino-4-methylcoumarin. Sodium sulfide (Na_2_S, Dojindo), an H_2_S donor, and each sample were added to 96-well black plates, mixed, and incubated at 37 °C for 6 h. To minimize H_2_S volatilization during the reaction, the plates were sealed with tape as described by Olson et al. [32]. Fluorescence measurements for AzMC and SSP4 were performed on a SpectraMax i3x microplate reader using the excitation/emission settings of 365/450 nm and 482/515 nm, respectively, following the manufacturer’s instructions. All samples and reagents were prepared in phosphate-buffered saline.

### 4.10. Statistical Analysis

All statistical analyses were performed using GraphPad Prism version 9 for Windows (GraphPad Software, San Diego, CA, USA). Values are expressed as mean ± standard deviation (SD). Statistical analysis was performed using one-way ANOVA followed by Dunnett’s multiple comparison test for statistical comparisons among groups, with *p* < 0.05 indicating significance. The results of active ingredient screening were evaluated using analysis of variance (ANOVA) and Tukey’s test, with *p*-values < 0.05 indicating significant differences.

## 5. Conclusions

In summary, the extract obtained from the silver tips (leaf buds) of *Camellia sinensis* markedly promoted supersulfide production in keratinocytes. Silver tip extract is rich in polyphenols, and its constituents EGC, ECG, and EGCG significantly enhanced intracellular persulfide biosynthesis. Among these, EGCG, which possesses both a galloyl group and a pyrogallol structure, exhibited the strongest capacity to induce supersulfide synthesis in keratinocytes. Mechanistic studies revealed that silver tip extract, with ECG and EGCG as its active components, promotes supersulfide production by regulating NRF2, xCT, CARS2, CBS, and CSE. Furthermore, the extract and these compounds catalytically oxidized H_2_S to reactive sulfur species, forming supersulfides through chemical reactions. Collectively, our findings indicate that the use of silver tip extract represents a novel anti-aging approach targeting supersulfide metabolism in the skin.

## Figures and Tables

**Table 1:** Contents of compounds in the silver tip extract and dried silver tip samples.

Compound	Silver Tip Extract (mg/g)	Dried Silver Tip (mg/g)
Caffeine (CF)	224.3	88.0
(+)-Catechin (C)	11.0	4.3
(−)-Epicatechin (EC)	9.5	3.7
(−)-Epigallocatechin (EGC)	20.2	7.9
(−)-Epicatechin gallate (ECG)	56.6	22.2
(−)-Epigallocatechin gallate (EGCG)	110.7	43.4

## Data Availability

The original contributions presented in this study are included in the article. Further inquiries can be directed to the corresponding author.

## Abbreviations

The following abbreviations are used in this manuscript:

SSP4	sulfane sulfur probe 4
CARS2	cysteinyl-tRNA synthetase 2
CBS	cystathionine β-synthase
CSE	cystathionine γ-lyase
MPST	3-mercaptopyruvate sulfurtransferase
NRF2	nuclear factor E2-related factor 2
CysSSH	cysteine persulfide
CysSSSCys	cysteine trisulfide
GSSH	glutathione persulfide
GSSSG	glutathione trisulfide
EGCG	(−)-epigallocatechin gallate
ECG	(−)-epicatechin gallate
NHEKs	Normal human epidermal keratinocytes
qPCR	Quantitative polymerase chain reaction
HPLC	High-performance liquid chromatography
EGC	(−)-epigallocatechin
C	(+)-catechin
CF	caffeine
EC	(−)-epicatechin
PDA	Photodiode Array
AzMC	7-azido-4-methylcoumarin
SD	standard deviation
